# Through the eyes of the student: Best practices in clinical facilitation

**DOI:** 10.4102/curationis.v40i1.1787

**Published:** 2017-08-28

**Authors:** Immaculate S. Muthathi, Catherine H. Thurling, Susan J. Armstrong

**Affiliations:** 1 Department of Nursing Education, University of the Witwatersrand, South Africa

## Abstract

**Background:**

Clinical facilitation is an essential part of the undergraduate nursing curriculum. A number of studies address the issue of clinical facilitation in South Africa, but there remains a lack of knowledge and understanding regarding what students perceive as best practice in clinical facilitation of their learning.

**Objective:**

To determine what type of clinical facilitation undergraduate students believe should be offered by clinical facilitators (nurse educators, professional nurses and clinical preceptors) in the clinical area in order to best facilitate their learning.

**Method:**

A qualitative, exploratory and descriptive study was conducted. Purposive sampling was performed to select nursing students from the second, third and fourth year of studies from a selected nursing education institution in Johannesburg. The sampling resulted in one focus group for each level of nursing, namely second, third and fourth year nursing students. Interviews were digitally recorded and transcribed verbatim, thematic data analysis was used and trustworthiness was ensured by applying credibility, dependability, confirmability and transferability.

**Main findings:**

The data revealed that participants differentiated between best practices in clinical facilitation in the clinical skills laboratory and clinical learning environment. In the clinical skills laboratory, pre-contact preparation, demonstration technique and optimising group learning were identified as best practices. In the clinical learning environment, a need for standardisation of procedures in simulation and practice, the allocation and support for students also emerged.

**Conclusion:**

There is a need for all nurses involved in undergraduate nursing education to reflect on how they approach clinical facilitation, in both clinical skills laboratory and clinical learning environment. There is also a need to improve consistency in clinical practices between the nursing education institution and the clinical learning environment so as to support students’ adaptation to clinical practice.

## Introduction

Nursing is a practice-based profession and the importance of clinical teaching in nursing education cannot be over-emphasised. Gaberson and Oermann (2010:8) state that clinical teaching is the most important component of nursing education, more so than classroom teaching and it is therefore regarded as an integral part of the undergraduate nursing curriculum. Scholars support the use of clinical facilitation as a vehicle to achieve the clinical teaching component of undergraduate nursing education (Needham, McMurray & Shaban 2016:138). Clinical facilitation aims to assist student nurses to apply the theory of nursing in real-life situations integrating theoretical knowledge and practical skills (Needham et al. 2016:138). Ensuring best practice in clinical facilitation is therefore vital to nursing education, as it is through clinical facilitation that students learn the skills and values of the nursing profession (Ali 2012:15).

Nursing students in South Africa are required to undergo a four year fulltime degree or diploma programme in order to qualify as a professional nurse. During this training programme, nursing students rely on clinical facilitation to maximise the time spent in the clinical learning environment to learn and perfect the art and skill of nursing (Okoronkwo et al. 2013:63).

In South Africa the role of clinical facilitation is a shared responsibility between nurse educators, professional nurses and clinical preceptors. According to the South African Nursing Council (SANC), nurse educators need to develop nursing student’s cognitive, psychomotor and affective skills and this may be achieved through effective clinical facilitation (SANC 2014:3). A professional nurse has a responsibility and accountability for the quality of care given to patients and as a result she has a moral duty to teach, mentor and supervise nursing students during their placement in her unit, in order to ensure students can deliver quality care to patients and ensure patient safety (Bruce, Klopper & Mellish 2011:256). The SANC requires that the clinical facility where students are placed for their clinical training provides learning opportunities that meet the needs of learners placed in these clinical practice areas, through clinical facilitation (SANC 2013:5). As a result, some clinical facilities employ clinical preceptors to assume a clinical facilitation role.

The incongruency in the roles of the nurse educator, professional nurse and clinical preceptor allows for shifting of responsibilities and often results in a sub-optimal quality of clinical facilitation. Rikhotso, Williams and De Wet (2014:1) also noted that challenges caused by the shifting of the clinical facilitation role between professional nurses and nurse educators can negatively impact students’ clinical learning.

Achieving effective clinical facilitation of undergraduate nursing students’ clinical learning in South Africa remains an ongoing challenge. Scholars allude to poor accompaniment of students by nurse educators as an inhibiting factor to clinical learning (Beukes, Nolte & Arries 2010:5). Some studies confirm lack of communication between nurse educators and professional nurses in the clinical learning environment as resulting in poorly planned clinical experiences for the students (Mochaki 2009:121; Rikhotso 2010:38). Research studies from various Nursing Education Institutions (NEIs) within the South African context support the evidence that there is dissatisfaction experienced by nursing students related to clinical facilitation (Magobe, Beukes & Müller 2010:3; Sibiya & Sibiya 2014:1943). In 2013, the National Department of Health reported that there is inadequate clinical facilitation of nursing students in the majority of clinical facilities in the country, resulting in newly qualified nurses being poorly prepared for their roles (National Department of Health 2013:21–22).

Scholars who advocate a student centred teaching approach in nursing education espouse the need to consider student’s perceptions and preferences of teaching and learning (Newton et al. 2012:2331). An assessment of students’ learning preferences and perceptions of best practice is envisaged to assist an educator to better view clinical facilitation through the eyes of nursing students as it supports a student centred teaching approach (Chiang, Chapman & Elder 2010:816; Hallin 2014:1444). Although a number of studies address clinical facilitation of nursing students in South Africa (Magobe et al. 2010:3; Sibiya & Sibiya 2014:1943), little is known about what nursing students perceive as best practices in clinical facilitation.

### Problem statement

In order for nursing education to be student centred, it is important that the student’s perceptions of clinical facilitation are considered, but little is known about what undergraduate students believe in this regard.

## Aim of the study

The aim of this research was to explore best practices in clinical facilitation from the student nurses’ perspective.

## Research objective

The study’s objective was to determine what type of clinical facilitation undergraduate students believe should be offered by clinical facilitators (nurse educators, professional nurses and clinical preceptors) in the clinical area in order to best facilitate their learning.

## Definition of key concepts

**Best practices** are the actions and ideas that represent the most efficient, effective or prudent course of action (Cambridge University Press 2014). In this study the term best practice will refer to all the actions performed by clinical facilitators that represent the most efficient, effective and prudent action as viewed by the nursing students during their clinical learning experience.

**Clinical facilitation:** is the process of helping and guiding students individually or in groups to work effectively to achieve their learning outcomes in their clinical learning environment and clinical skills laboratory; it involves planning activities that may stimulate and support their learning (Bruce et al. 2011:112).

**Clinical facilitators:** in this study, refer to all nurse educators, professional nurses and clinical preceptors who facilitate learning of the undergraduate nursing students in the clinical practicum.

**Clinical learning environment:** in this study refers to the clinical setting where students can learn and develop clinical nursing skills in a real-life situation, under the guidance of an experienced professional.

**Clinical skills laboratory:** is ‘an educational facility in which a learner can be taught and be able to practice clinical skills before using them in clinical settings’ (Al-Elq 2007:2). In this study, this refers to the simulation laboratory in a NEI where nursing skills are demonstrated and practised in a relatively safe environment.

### Contribution to the field: Significance of the study

The findings of this study add knowledge to the nursing profession in regard to understanding nursing students’ perceptions of best practice facilitation of their clinical learning. It also provides recommendations for clinical facilitators as to how best to enhance clinical facilitation in the undergraduate student’s clinical learning experiences.

## Research design and method

### Design

A qualitative, exploratory and descriptive study was conducted. Qualitative design is suitable for this study because it is a method of enquiry focusing on the in-depth aspects of the meaning and opinions of these selected participants and aims at understanding a phenomenon (best practices in clinical facilitation) from their perspective (Botma et al. 2010:81–82). An exploratory design is undertaken when little is known about the phenomenon under study (Botma et al. 2010:185). In this study it aimed to explore the full nature of the phenomenon, its manifestation and the factors that relate to it. Descriptive design aimed to describe characteristics of the phenomenon and to provide the reader with a picture of the situation as it naturally happens (Botma et al. 2010:110).

### Population and sampling

The population consisted of nursing students studying for an undergraduate degree leading to registration as a nurse (general, community and psychiatric) and midwife at a selected NEI. One hundred and eight undergraduate nursing students (*N* = 108) were registered in this NEI for the Nursing Degree in 2014: 8 first year students, 29 second year students, 14 third year students and 17 fourth year students. Purposive sampling was employed, first year nursing students were excluded as they had little exposure to clinical practicum and therefore would not be able to judge best practices. Second, third and fourth year nursing students were included as they had adapted to their clinical learning environment and were assumed to have an ability to describe their perceived best practice. All eligible students were invited to participate in the study. The realised sample consisted of 24 participants (*n* = 24), who participated in three focus groups: 8 second year, 7 third year and 9 fourth year nursing students, respectively. The sample size was determined with data saturation being achieved.

### Data collection method

The focus group method of data collection was used as this is useful when the researcher is exploring perceptions and looking for various ideas (Greeff 2005:287). Focus groups allowed the researcher to explore different views from a number of participants, with the aim of merging ideas from the groups. One focus group interview was conducted for each year group. This allowed the researcher to view similarities and differences in perceptions per group.

The researcher conducted the focus groups, assisted by an experienced field worker writing field notes to be used to validate the transcribed data. All focus group interviews were digitally recorded and conducted at a time and place convenient to the participants. During the focus group interview sessions, a circle seating arrangement was used to encourage a sense of equality amongst participants and interviewer. An interview guide, developed by the researcher, was used to ensure that different focus groups were conducted in the same manner and to focus the discussion within the objectives of the interview (Krueger & Casey 2015:37). Introduction was done, rapport was developed with the focus group participants and ground rules were set. Information letter was re-read and consent forms were signed. Key concepts such as clinical facilitation, best practice and clinical practicum were explained and defined for the participants, to make sure they understood the key concepts of the discussion. The central question that guided the focus group discussion was ‘As student nurses what do you think constitutes best practice in your clinical practicum experience with regard to clinical facilitation?’ Communication clarification techniques, such as probing, summarising, paraphrasing, listening, reflecting, using minimal verbal response and focusing, were used to ensure detailed exploration of the students’ perceptions of best practices. Data collection was discontinued after data saturation had been reached as evidenced by no new information being presented by the participants. The duration of the focus group interviews was between 55 min and 60 min.

### Data analysis

All digitally recorded focus group interviews were transcribed verbatim by the researcher. The researcher listened repeatedly to the recorded data, becoming immersed in the data and gaining the overall view of the meaning of the data. Field notes were read while listening to the recording to ensure consistency of the data. Creswell’s method of thematic data analysis was used to identify patterns of meaning across a data set (Creswell 2014:194–196). Transcribed data were read and analysed across all interviews, reflecting on its meaning. The underlying meanings were written on the side of the margin. The participant’s perceptions which described best practice in clinical facilitation were noted and highlighted across all interviews. Perceptions that occurred frequently in each focus group were noted and underlined, forming themes. Themes were then analysed for each focus group and across the other focus groups. The researcher and co-coder analysed and coded the data and discussed the most prominent findings of the study. The participants were approached to member check the findings and validate the truth of the coded subthemes.

### Context of the study

The context for this study was a NEI associated with an accredited academic hospital in Johannesburg, where the undergraduate nursing students of the selected NEI were placed for their clinical learning. The nursing students’ clinical facilitation in this hospital was overseen in the following manner: The nurse educators employed by the NEI, as well as the professional nurses employed by the hospital, and clinical preceptors employed by either a NEI or a hospital were expected to assume a clinical facilitation role.

### Ethical consideration

Permission to conduct the study was obtained from the university’s Postgraduate Research Committee and Human Research Ethics Committee (Medical; M140382). Permission to invite nursing students to participate in the research study was sought and granted from the Head of the Nursing Department at the selected NEI at which participants were registered as student nurses. Participants gave informed consent for voluntary participation and for audio recording of the interviews, after thorough explanation of the nature and extent of the study by the researcher. Participant numbers were used to ensure anonymity. The audio recordings were kept under lock and key by the researcher after she completed verbatim transcription of the interviews; this was to ensure confidentiality. No one except the researcher and her supervisor had access to the data.

## Trustworthiness

In this study the evaluation of data quality was ensured by using the method of trustworthiness described by Lincoln and Guba (1985:290), which includes credibility, dependability, confirmability and transferability. Credibility was ensured by planning the study with two senior researchers, use of an interview guide to ensure questions are focused on the research objectives and the use of field notes to validate the transcribed data. Independent data coding was done by the researcher and her co-coder and member checking was performed with participants to validate the truth of the resultant themes. Transferability was ensured through a detailed description of the research methodology, the research context and purposive sampling. Dependability was ensured by providing a detailed description of the research methodology. Confirmability was assured by having both the researcher and her supervisor analyse and code data independently, as well as confirming results with the participants, audio recording of interviews and record keeping for the audit trail.

## Findings and discussion

Two main themes concerning best practices in clinical facilitation of undergraduate nursing students emerged with six subthemes as displayed in Table 1. Subthemes are described with participants’ voices indicated in italics.

**TABLE 1:** Themes and subthemes.

Themes	Subthemes
1. Best practices in facilitating learning in the clinical skills laboratory	1.1. Pre-contact preparation 1.2. Technique of demonstration 1.3. Optimising group learning
2. Best practices in facilitating learning in the clinical practicum	2.1. Standardisation of procedures 2.2. Allocation to clinical areas 2.3. Support for students

*Source*: Muthathi 2015:40

### Theme one: Best practices in facilitating learning in the clinical skills laboratory

Three actions that are described as subthemes were identified under this theme namely: **pre-contact preparation, technique of demonstration** and **optimising group learning.**

#### Subtheme one: Pre-contact preparation

To some participants, best practice meant being informed about which procedure is to be demonstrated in the coming simulation session. This is evident in the following quote:

‘Ok, before you get to the Dem room [*Clinical Skills Laboratory*], it’s good that we have paper telling you which skills that you going to do before we get to the class, that’s good.’ (Participant 1, Female, 2nd year)

‘ … With the skills for the Dem room [*Clinical Skills Laboratory*], if we may be given notes first, show the demonstration later, like what (name of the lecture) did when she taught us on suturing, like give lecture first.’ ( Participant 2, Female, 2nd year)

Pre-contact preparation by the nurse educator, such as sending notes and guidelines regarding the procedure prior to the simulation session was deemed best practice and allowed participants to prepare for simulation. This idea is supported by Bruce et al. (2011:268) who believe students must have information about the procedure to be demonstrated; specifically, they must be aware of the crucial points to note during demonstration. Helgesen, Gregerson and Roos (2016:1) also support preparedness as an important factor in student’s clinical learning. Although the literature supports the notion of pre-contact preparation, not all the students prepare for clinical lessons when provided with resources to do so.

#### Subtheme two: Technique of demonstration

There were two points of view about what the participants considered best practice in skills demonstration. To some students, best practice was to see the whole procedure demonstrated without any discussion, and then to discuss it later. Some students believed that the most prudent action was for the clinical facilitator to break down the complex procedure into small manageable portions. The following comments illustrate the contradictory views of the participants:

‘It’s better you do the skill [*procedure*], do the whole skill [*procedure*] with no one distracting or asking questions, or comments, do the skill completely and talk later about the skill.’ (Participant 1, Female, 2nd year)

‘So they [*clinical facilitators*] will do one section, then I’ll follow through that, then they will do the next session and I’ll follow through that and the next session and then I’ll follow through on that. Not all in one go and then expect me to do it. Rather do it in step by step.’ (Participant 1, Female, 3rd year)

The above-mentioned narrative depicts disagreements on what students considered as best practice in demonstration of a nursing procedure. According to Kharb et al. (2013:1090), students have different learning styles, which should be considered during teaching sessions. Some individuals prefer to process information in a step by step format, while others prefer to see the bigger picture before they can master the details (Kharb et al. 2013:1090).

Learning styles refer to the individual’s approach on how they take in, organise and process information depending on their own strengths and preferences (Kharb et al. 2013:1090). As a result, when considering differences in student learning styles, the researcher believes it could be effective to demonstrate the procedure as a whole, first to show the overview and then demonstrate it in small components allowing students to practise and master the small components before they perform the whole procedure.

#### Subtheme three: Optimising group learning

Most participants suggested that the best practice in demonstrating a skill is achieved when students are divided into small groups in order to increase their ability to observe the demonstration and to allow students a chance to practise the procedure and take an active part in the learning experience. This is noted in the following comment:

‘To have the facilitators either split us into smaller groups, this is how we will get to learn more in practical, because when we have twenty people all watching, some people can’t see, some people get distracted. So it’s smaller groups or one-on-one.’ (Participant 2, Female, 2nd year)

Most participants agreed that they preferred small-group learning for clinical demonstrations, for best practice to be achieved in the clinical skills laboratory, to ensure that they are all able to see the procedure and are able to have an opportunity to practice and repeat the demonstrated procedure. These students’ perceptions are supported by the findings in a study by Bray (2014:30–34) on student views of their clinical learning experiences. Bray found that a high student to facilitator ratio per clinical demonstration session was associated with poor learning outcomes, while small-group teaching facilitated learning. Shortages of clinical facilitators are an obstacle to small-group learning and are exacerbated by a lack of collaboration between the nurse educators, professional nurses and clinical preceptors. A possible answer to this issue is a team approach in clinical facilitation, bringing together nurse educators, professional nurses and clinical preceptors and including involvement of postgraduate nursing students to be actively involved in clinical teaching of undergraduate nursing students.

### Theme two: Best practices for facilitating learning in the clinical practicum

Three subthemes were identified as best practices in facilitating learning in the clinical practicum under this theme, namely**:** *standardisation of procedures, allocation to clinical areas* and *support for students*.

#### Subtheme one: Standardisation of procedures

Most students across all focus groups described best practice in clinical facilitation as a consistency in performing procedures between the clinical skills laboratory and the clinical facility. They said consistency reduces confusion when transferring skills learned in clinical skills laboratory to clinical practicum. This is confirmed in the following comment:

‘Best practice facilitation will be, whatever skill we learn in the department [*Clinical Skills Laboratory*] we actually do the very same process in the wards, because sometimes there is a big discrepancy, we learn it this way in the skills laboratory, and when we get into the ward the sisters do it differently.’ (Participant 1, Male, 3rd year)

Standardisation of procedures between clinical learning environment and clinical skills laboratory took centre stage across all the interviews conducted in this study. Participants emphasised the need for standardising the performance of procedures. They mentioned that there is a discrepancy on how procedures are performed in the clinical skills laboratory and in different wards in the clinical facility, which leads to confusion for them as learners. Increased workloads coupled with staff shortages are associated with a lack of compliance to good quality standards of practice (Magobe et al. 2010:4). Msiska, Smith and Fawcett (2014:38) also state that a lack of clinical equipment and other resources leads to the need to improvise and to poor nursing practices. These shortcuts, together with improvised procedures, affect the quality of clinical learning. Msiska et al. (2014:38) also say that in such situations nursing students are left confused and lacking confidence in performing skills because they learn procedures in a perfect environment, which is well-equipped such as the clinical skills laboratory and are then placed in an often imperfect and poorly resourced clinical facility. The researcher believes that basic nursing skills should be demonstrated both in the clinical skills laboratory and in the clinical practicum. Nurse educators must discuss with students the possible acceptable means of improvising and emphasise the principles that ensure infection control and patient safety.

#### Subtheme two: Allocation to clinical practicum

Another issue that was emphasised as best practice was the length of time of students’ clinical placements and the alignment of the placement to theory. Full day allocation in the clinical practicum was deemed best practice as opposed to half day placements.

‘You really cannot learn much from 7 am to 1 pm, … you have four days in a month to go to that specific ward for six hours per day and you get there, they expect us to know our patients … [*if*] we work seven to seven and we get enough exposure, I feel that for something to be regarded as achieved best practice it would be good if we had more exposure.’ (Participant 1, Male, 2nd year)

Best practice was also associated with being allocated to the clinical practicum soon after learning in the clinical skills laboratory, so as to reinforce the demonstrated skills and to facilitate the skills transfer to the real situation. One of the participants had this to say:

‘ … It would help if the demonstrations we’ve got in the dem room [*Clinical Skills Laboratory*] and then the allocations that we have got in the clinical setting, like correlated with each other. Because it doesn’t help you teach me something in Jan or you demonstrate something in January, but you are only going to allocate me to that particular ward let’s say in May/June. Whatever I was taught then, it’s still there but it’s not as fresh.’ (Participant 4, Female, 4th year)

The Nursing Education Stakeholders Group (2012:5) supported the findings that when planning a student’s allocation to clinical practice, it is important that the continuity of learning is ensured through the alignment of the practical placement to the related theory. Lack of suitable clinical placement sites leads to competition for clinical placement areas, and as a result, it is common not to have the students’ placement aligned to the theory discussed in the classroom.

It is the researcher’s opinion that this issue is interrelated with lack of standardisation of procedures, which leads to student’s worrying that by the time they are placed in the clinical facility, they would have forgotten how the procedure was demonstrated in the clinical skills laboratory. If the performance of procedures in both the clinical facility and the clinical skills laboratory were to be standardised, students would know that they would still observe the similar procedure in the clinical practicum and easily recall what was taught in the clinical skills laboratory.

Being allocated for a full day (12 h) per week was suggested by the nursing students to influence best practice. In the context in which the study took place, students were only placed for half day (6 h) per week because of academic commitments to non-nursing courses.

Levett-Jones et al. (2008:11–12) studied the relationship between the duration of clinical placement and the student’s feeling of belonging during their clinical placement. The findings of their study revealed that during the first 2–4 weeks’ placement, students undergo a phase of adjusting to the clinical placement area. During this phase, the students focus less on achieving their learning objectives, as they are overwhelmed with doubt and nervousness about their clinical placement. Therefore, Levett-Jones et al. (2008:14) believe that allocating students for less than four weeks in one clinical area is not considered best practice, as extended placements promote belongingness in the students, which then enables self-directed participation in relation to their clinical learning. However, in the same study it was found that placing students in a negative practice environment for an extended period was not valuable to student learning (Levett-Jones et al. 2008:14–15).

In contrast, Gilmour et al. (2013:e21) found that the quality of student learning in the clinical environment depends on the effectiveness of the educator whom the student is paired with, rather than the length of exposure. The researcher believes the best possible solution to ensure students achieve their objectives is for the nurse educator to be available during the 6 h of student allocation, supporting them and assessing competencies. Nurse educators should choose placements that promote student learning socialisation.

#### Subtheme three: Support for students

The students also emphasised the importance of active involvement of nurse educators in clinical teaching during their clinical practicum as a means of support to them. They verbalised challenges of transferring skills to the real-life situation and believed the person who teaches in the nursing skills laboratory should be the same person who assists them to adapt the learned skill in the clinical practice area. The following comments confirm this finding:

‘Best practice would be, to have the facilitator to teach the skill in the ward on an alive human being.’ (Participant 7, Female, 2nd year)

‘… If you have a clinical facilitator that taught you theoretically and in the Sim Lab [*Clinical Skills Laboratory*] and go with you in the hospital, … You enforce principles there once you’ve worked with him in the hospital then should someone else comes and work with you, you know you can show them the right way.’ (Participant 1, Female, 3rd year)

The students stated that the presence of the nurse educators in the clinical area alleviates confusion caused by the lack of standardisation of procedures as well as for the nursing student’s need for support relating to transferring skills learned in the clinical skills laboratory to real-life situations.

It was interesting to note that all nursing students, irrespective of the level of their training, verbalised a need for the support and supervision by nurse educators in the clinical areas. The published literature supports the fact that supervision, support and positive feedback increases the student’s confidence in implementing nursing skills in real-life situations and that the nurse educator is seen as a guide and a confirmer of the attainment of intended learning goals (Houghton et al. 2013:1965; Tiwaken, Caranto & David 2015:70). The findings of this study show that nurse educators must be closely involved in clinical facilitation.

### Practical implications of the study

This study explored the perceptions of student nurses at a university, and while their point of view is valid, some of the recommendations are not feasible in the current economic climate, where there is a shortage of professional nurses and nurse educators. This has been exacerbated by the demand to increase the student numbers at institutions of higher education but no provision has been made to increase the nurse educator, professional nurses and clinical preceptor posts accordingly. In this research, students were asked to explore clinical facilitation best practices. Understandably, the recommendations were made based on the ‘ideal situation’ rather than the realities and constraints of providing clinical facilitation.

The discrepancies in practice between nursing skills laboratory and clinical learning environment depicts that achieving best practice in clinical facilitation remains a challenge that requires collaborative interventions by nurse educator, professional nurse and clinical preceptor. To address the clinical disconnect between the skills taught in the nursing skills laboratory and in the clinical learning environment, all professional nurses should be encouraged to have a joint commitment to the clinical environment and to academia.

## Limitations

The setting of this study was one selected NEI and an academic hospital where the participants were placed for their clinical practicum; therefore, the findings of this study cannot be generalised to the perceptions of other nursing students in a different context. However, the findings may be transferable with consideration of the study context.

## Recommendations

The following recommendations were developed based on the findings of this study:

- Online learning platforms should be used to keep nursing students informed of their clinical requirements and other information needed on a real-time basis. Clinical skills can be recorded and made available to students to access on the student learning online portal.
- A team approach in clinical facilitation is required bringing together professional nurses, nurse educators and clinical preceptors and including involvement of postgraduate nursing students in clinical teaching of undergraduate nursing students. Innovative funding options should be explored to support the initiative of joint posts as well as postgraduate preceptor posts. The Clinical Training Grant should be used to fund nursing education to a greater extent to improve student: facilitator ratios.
- Clinical education workshops should be conducted on an ongoing basis, aiming at revising clinical nursing procedures to share knowledge between clinical facilitators. This intervention will ensure evidenced-based practices and the standardisation of performance of procedures in both the clinical skills laboratory and in clinical facilities. This might encourage professional nurses to teach in the clinical learning environment, as they will feel supported and more involved in the student’s development. These workshops will promote collaboration between all clinical facilitators and allow for constructive discussion of clinical facilitation challenges experienced by facilitators and improvement methods.
- Clinical placements and the theoretical component of the nursing curriculum should be considered and where possible aligned. Whereas it is acknowledged that it is often difficult to teach university students using a block programme, wherever possible this should be considered in order to improve theory and practice correlation.

## Conclusion

The findings of this study suggest a need for all nurses involved in clinical facilitation to reflect on how they plan and conduct their clinical facilitation in the clinical skills laboratory and in the clinical learning environment. Nurse educators should also be closely involved in facilitating learning in the clinical learning environments to assist in theory–practice integration. Collaboration between nurse educators, professional nurses and clinical preceptors for quality clinical facilitation is necessary.
